# Optimized NGS Approach for Detection of Aneuploidies and Mosaicism in PGT-A and Imbalances in PGT-SR

**DOI:** 10.3390/genes11070724

**Published:** 2020-06-29

**Authors:** Carmen M. García-Pascual, Luis Navarro-Sánchez, Roser Navarro, Lucía Martínez, Jorge Jiménez, Lorena Rodrigo, Carlos Simón, Carmen Rubio

**Affiliations:** 1 R&D Department, Igenomix, 46980 Valencia, Spain; luis.navarro@igenomix.com (L.N.-S.); roser.navarro@igenomix.com (R.N.); lucia.martinez@igenomix.com (L.M.); jorge.jimenez@igenomix.com (J.J.); lorena.rodrigo@igenomix.com (L.R.); carlos.simon@igenomix.com (C.S.); carmen.rubio@igenomix.com (C.R.); 2 Igenomix Foundation, 46980 Valencia, Spain; 3 School of Medicine, University of Valencia/INCLIVA, Valencia 46106, Spain; 4 Department of Obstetrics and Gynecology, School of Medicine, Stanford University, Stanford, CA 94305, USA; 5 Department of Obstetrics and Gynecology, Baylor College of Medicine, Houston, TX 77030, USA

**Keywords:** NGS, aneuploidy, mosaicism, segmental, translocations, PGT-A

## Abstract

The detection of chromosomal aneuploidies and mosaicism degree in preimplantation embryos may be essential for achieving pregnancy. The aim of this study was to determine the robustness of diagnosing homogenous and mosaic aneuploidies using a validated algorithm and the minimal resolution for de novo and inherited deletions and duplications (Del/Dup). Two workflows were developed and validated: (a,b) preimplantation genetic testing for uniform whole and segmental aneuploidies, plus mixtures of euploid/aneuploid genomic DNA to develop an algorithm for detecting mosaicism; and (c) preimplantation genetic testing for structural rearrangements for detecting Del/Dup ≥ 6 Mb. Next-generation sequencing (NGS) was performed with automatic library preparation and multiplexing up to 24–96 samples. Specificity and sensitivity for PGT-A were both 100% for whole chromosomes and segmentals. The thresholds stablished for mosaicism were: euploid embryos (<30% aneuploidy), low mosaic (from 30% to <50%), high mosaic (50–70%) or aneuploid (>70%). In the PGT-SR protocol, changes were made to increase the detection level to ≥6 Mb. This is the first study reporting an accurate assessment of semiautomated-NGS protocols using Reproseq on pools of cells. Both protocols allow for the analysis of homogeneous and segmental aneuploidies, different degrees of mosaicism, and small Del/Dup with high sensitivity and specificity.

## 1. Introduction

Aneuploidies underlie most reproductive failures in humans [1] and, based on large datasets, over half of the embryos produced through in vitro fertilization (IVF) are aneuploid [2,3]. Thus, preimplantation genetic testing for aneuploidy (PGT-A) was proposed to improve pregnancy rates per transfer and to decrease miscarriage mostly in advanced maternal age (AMA) patients [4,5,6,7]. Recently, PGT-A has been shown to offer shorter time for pregnancy with lower cost compared to conventional IVF for some subgroups of couples [8,9]. Currently, PGT-A includes the study of uniform aneuploidies, small deletions/duplications (Del/Dup ≥ 10 Mb), and mosaicism. Preimplantation genetic testing for structural rearrangements (PGT-SR) aims to detect smaller imbalances (≥6 Mb), mostly in embryos where at least one parent is a carrier of a balanced translocations and/or inversions.

The earliest technologies to assess all 24 chromosomes were comparative genome hybridization arrays (aCGH), single-nucleotide polymorphism (SNP) microarrays, and quantitative polymerase chain reaction (qPCR). These were applied to both PGT-A [10,11,12,13] and PGT-SR [14,15]. More recently, techniques have been developed based on next-generation sequencing (NGS). NGS has significant advantages. It is a versatile platform that can be used for detecting uniform/whole aneuploidies and Del/Dup [16] and, compared to aCGH, is a reliable high-throughput technology with higher resolution and a broader dynamic range facilitating mosaicism diagnosis [15,17]. It is also cheaper and requires less hands-on time. Current NGS protocols consist of (a) whole genome amplification (WGA) and barcoding; (b) library preparation, purification, and templating; (c) loading and sequencing; (d) alignment of sequenced reads to a human reference genome; and, finally, (e) data analysis and reporting. The templating preparation steps, chip loading, and data analysis can potentially be automated, which is strongly recommended to decrease technical and human errors and increase the robustness and reproducibility of results when processing large numbers of samples. 

However, two important issues should be addressed before implementing NGS in a clinical diagnostic laboratory: (1) defining the sequencing parameters required for each application, the minimal resolution of each platform to detect Del/Dup and identify the presence of mosaicism; and (2) creating a bioinformatics pipeline and diagnostic algorithms best able to avoid the subjectivity linked to the visualization of sequencing plots. Del/Dup detection is limited by the minimal resolution of the platform, number of reads and signal/noise ratio affecting the minimal fragment size that can be detected [18,19,20]. Mosaicism detection is challenging since the degree of mosaicism is estimated from a single trophectoderm biopsy (TE) with an uncertain number of cells. As live births after the transfer of mosaic embryos have been reported [20,21,22,23] and the clinical outcome seems to be influenced by the level of mosaicism [24], the identity [25] and, the number [26] of affected chromosomes; a proper validation to define mosaicism thresholds is required for each platform to avoid overdiagnosis due to technical artefacts.

To address these issues, we sought to validate a semi-automated NGS protocol for PGT-A to detect uniform whole-chromosome aneuploidies and segmental aneuploidies ≥10 Mb, and a modified protocol to increase the resolution up to 6 Mb to detect imbalances in carriers of structural rearrangements (PGT-SR). Regarding mosaicism, we wanted to define thresholds for an accurate diagnosis and develop an algorithm to automatically detect its levels in TE samples, avoiding inter-individual and inter-laboratory subjectivity.

## 2. Materials and Methods 

### 2.1. Experimental Design

This study was carried out in two phases from August 2017 to April 2018. Phase I, for PGT-A, was conducted from August 2017 to February 2018 to validate the detection of uniform aneuploidies, Del/Dup ≥ 10 Mb, and mosaic whole chromosome aneuploidies. Phase II, for PGT-SR, was carried out from February 2018 to April 2018 for the detection of imbalances ≥ 6 Mb. In all validation experiments, the tests were assessed using cell lines/DNA samples purchased from the NIGMS Human Genetic Cell Repository at the Coriell Institute for Medical Research (Camden, NJ, USA). All cell lines were grown in cell culture conditions established by the manufacturer. Before being collected, cells were passaged once. Then, confluent cells were detached using Tryple E [27] and resuspended in PBS (Gibco, Walthan, MA, USA). Cells were isolated under a dissecting microscope and placed in sterile PCR tubes (Eppendorf, Hamburg, Germany). All samples were analyzed at least in triplicate.

**Figure 1:**
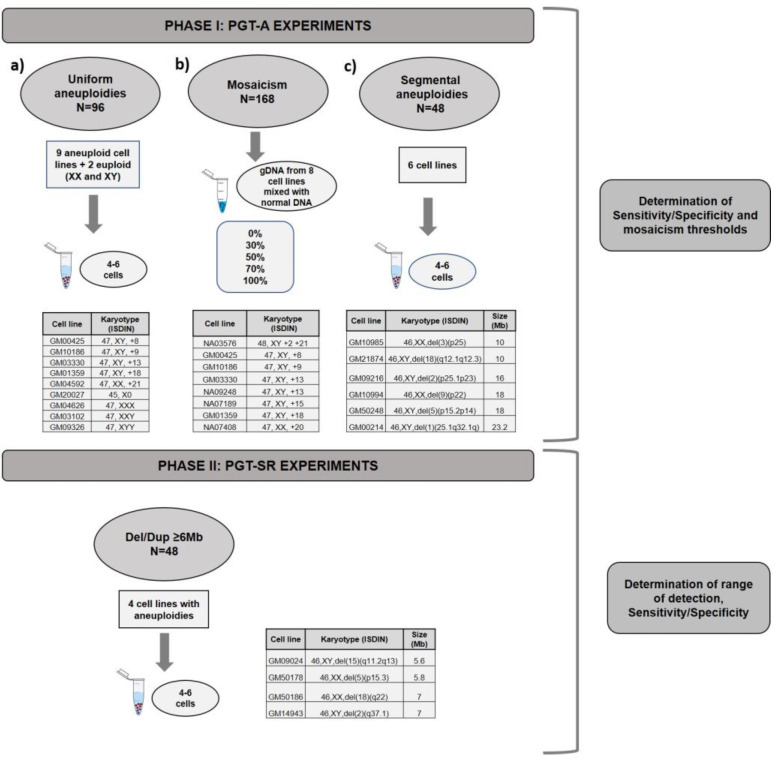
Experimental design for the validation of PGT-A and PGT-SR. Phase I: (**a**) PGT-A validation was divided in 3 experiments: (**a**) PGT-A for full aneuploidies, (**b**) PGT-A for mosaicism and, (**c**) PGT-A for segmental aneuploidies ≥ 10Mb. Phase II: PGT-SR for small rearrangements Del/Dupl ≥ 6Mb.

For phase I, three types of experiments were designed (Figure 1): (a) pools (*n* = 96 samples) of 4–6 cells mimicking TE biopsies from nine cell lines of known karyotype with aneuploidies in chromosomes 8, 9, 13, 18, 21, X0, XXX, XXY, and XYY as well as two with normal XX and XY karyotypes, hence 11 cell lines in total; and (b) mixes (*n* = 168 samples) of gDNA with different percentages of euploid (normal XX and normal XY) and aneuploid (0%, 30%, 50%, 70% or 100%) chromosomes (2, 8, 9, 13, 15, 18, 20 or 21). Once the algorithm was established, we tested its ability to correctly diagnose samples prepared to have 40%, 60%, or 100% mosaicism using two cell lines with trisomy in chromosome 8 or 9 (GM00425 and NA09287, respectively). To finalize the algorithm’s testing, we applied the algorithm retrospectively to 14,108 TE biopsies analyzed in 10 different diagnostic laboratories from our group and estimated the percentage of mosaicism in these clinical TE biopsies. 

To establish the algorithm’s ability to detect segmental aneuploidies ≥ 10 Mb, (c) pools (*n* = 48 samples) of 4–6 cells from six cell lines with Del/Dup ranging from 10 to 24 Mb were used. 

Phase II experiments were designed to validate PGT-SR for imbalances ≥ 6 Mb in pools (*n* = 48) of 4–6 cells using four cell lines from carriers of segmental aneuploidies with sizes from 5.6 Mb to 7 Mb (Figure 1). This protocol was optimized to increase resolution, as explained below. 

### 2.2. NGS Protocol for PGT-A and PGT-SR 

The NGS platform validated in this study was a semiautomated protocol using the Ion Chef™ equipment for library preparation and the S5 XL sequencer (ThermoFisher Scientific, Walthan, MA, USA). Samples were tested in batches of 24 or 96 (520 and 530 chips, respectively) for PGT-A and batches of 12 (520 chips) for PGT-SR (ThermoFisher Scientific). WGA and DNA barcoding were performed using the Ion ReproSeq PGS Kit (ThermoFisher), following the manufacturer´s instructions. The amplified DNA was purified, quantified with the Qubit™ (Qubit dsDNA HS Assay Kit ThermoFisher), and diluted to 80 pM before placing it in the Ion Chef™ equipment that automates preparation of the library and templates as well as chip loading, significantly reducing the hands-on time and interexperiment variability. The complete workflow from sample processing to reporting was completed in 12–14 h depending on the number of samples processed simultaneously. 

For PGT-SR, the original protocol was modified by doubling the number of reads per sample, loading the 520 chips with half of the samples (12 instead of 24). Purification steps were improved to increase DNA integrity, yield, and purity, allowing enrichment of the final library for fragments with the optimum length for the sequencer to read and increasing the quality of the sequencing. 

Quality parameters (QC) for both the entire run and individual samples were examined, with the most critical run parameters being loading percentage, live Ion Sphere Particles (ISPs) percentage, polyclonality and usable reads. The first factor impacting the average number of useful reads is the loading of the run, which indicates the number of chip wells containing ISP (with DNA (templated) or without (non-templated)). Templated ISPs, the ones that are sequenced, are termed ‘live’. Polyclonality refers to ISPs with more than one library template population (different DNA fragments). Each ISP should have only one DNA population, hence reads from ISPs with polyclonality are removed from analysis. The combination of these factors determines the usable read number, that will be divided among all samples in the run. Acceptable values for a run were: ≥70% loading, >98%, Live ISPs, <50% polyclonality, and >30% usable reads. For individual samples, the most important QC parameters were: the number of reads (required to be >70,000 for PGT-A and >120,000 for PGT-SR), the dispersion/noise of the profile as measured by the mean absolute percent deviation (MAPD) (required to be <0.3), and the number of duplicates (required to be < 30%). A sample was considered informative if these parameters were met.

### 2.3. Bioinformatics Analysis and Interpretation of Results 

Phase I: PGT-A for uniform whole chromosome and segmental aneuploidies ≥ 10 Mb and mosaicism

Sequencing data obtained by the S5 sequencer were processed and transferred to Ion Reporter software for data analysis. This software uses the bioinformatic tool ReproSeq w1.1 workflow to detect 24-chromosome aneuploidies from a single whole-genome sample with low coverage (minimum 0.01×). Normalization was done using the bioinformatics baseline ReproSeq Low-Coverage Whole-Genome Baseline generated from multiple normal samples. 

For all full/partial chromosomal regions detected by the software, we computed the difference value (DV) parameter, defined as DV=SNMC+CNMP×EP, where:

- Sample Normalized Mean Coverage (SMNC) is the observed ratio of reads in the sample;
- Control Normalized Mean Coverage for 1 copy (CNMP1) is the expected ratio of reads for one copy if the sample is normal;
- Expected Ploidy (EP) is the expected number of copies.

The DVs from all regions (positives for gains and negatives for losses) were used to establish the mosaicism and ploidy cutoffs according to the median values obtained in the different experiments that included uniform aneuploidy and different levels of mosaicism (0%, 30%, 50%, 70% or 100%). Different thresholds were defined to classify four levels of aneuploidy: euploid (<30% aneuploid), low-degree mosaicism (from 30 to <50% aneuploid), high-degree mosaicism (from 50 to <70% aneuploid) and aneuploid (≥70% aneuploid). In the pipeline for the diagnosis algorithm, all run and individual sample QC parameters were uploaded as well as the individual bam files, incorporating the aneuploidy classification described above. 

After validation and development, the algorithm was verified by calculating the incidence of mosaicism retrospectively in 14, 108 TE biopsies from 10 diagnostic laboratories (January–June 2019). The difference among the laboratories was studied using ANOVA.

Phase II: PGT-SR for Del/Dup ≥ 6 Mb

The analysis of these of samples was subjected to small changes in the workflow of the bioinformatic analysis. The confidence filter was lowered to increase sensitivity for smaller chromosome segments.

### 2.4. Evaluation of Efficiency, Concordance, Sensitivity and Specificity 

To determine the efficiency of the protocols, the percentage of informative samples was determined for each individual experiment. Sensitivity and specificity values were determined using only the informative samples (those meeting QC criteria). Concordance rates per sample were estimated as the percentage of samples showing the expected result according to the cell line karyotype. Sensitivity was defined as the percentage of samples showing the expected aneuploidy for each cell line and was calculated as True Positive ÷ (True Positive + False Negative). Specificity was defined as the probability of diagnosing a sample as euploid when there is no aneuploidy and it was defined as True Negative ÷ (True Negative + False Positive). 

## 3. Results

### 3.1. Phase I: PGT-A for Uniform Whole Aneuploidies, Mosaicism, and Segmental Aneuploidies (≥10 Mb)

For uniform whole-chromosome aneuploidies, 96 samples from 11 cell lines with known karyotype (2 of them normal XX and XY) were analyzed in one 530 chip sequencing run. All uniform samples met QC criteria and had perfect informativity and concordance rates (Table 1). The average of reads per sample was 173,053 (87,767–374,809; SD = 67,485) and the MAPD (Median Absolute Pair-wise Difference) that gives information about the noise of the profile was remarkable (0.17 (0.111–0.288; SD = 0.038). Importantly, no false negatives or positives were identified; hence, both sensitivity and specificity were 100%.

For segmental aneuploidies (Del/Dup ≥ 10 Mb), 48 samples from four cell lines were analyzed in two 520 chip runs. Again, all samples passed QC, and informativity and concordance rates were high as summarized in Table 1. On average, there were 126,780 reads (78,606–264,095; SD = 47,676) per sample and an MAPD value of 0.194 (0.151–0.234; SD = 0.031). Sensitivity and specificity were 100%.

For determining thresholds for different degrees of mosaicism, 168 gDNA samples from 10 different cell lines were analyzed in one 530 chip and three 520 chips. Informativity was 99.4% (167/168), mean number of reads 154,934 (75,598–290,805; SD = 38,562), and MAPD 0.180 (0.127–0.285; SD = 0.033).

**Figure 2:**
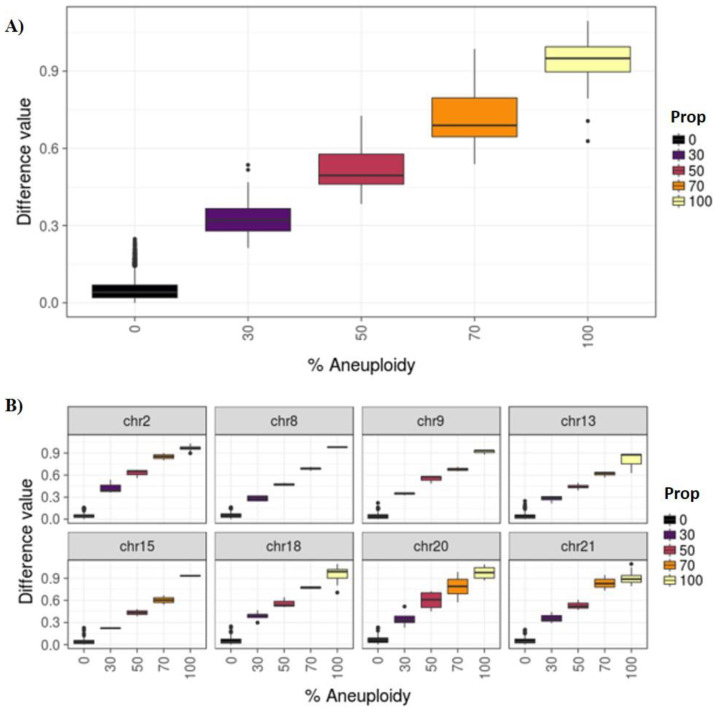
(**A**) Difference values for all chromosomes by known percentage of mosaicism. (**B**) All samples by selected chromosomes and proportion.

Four categories were established: euploidy (<30% aneuploid cells), low degree mosaicism (30–50%), high degree mosaicism (>50–70%), and aneuploidy (>70%). To classify samples in these categories, we defined the thresholds of different degrees of mosaicism. These thresholds were calculated using our cell line models to mimic different levels of mosaicism. The mean difference values for each category were 0.05 (SD = 0.04), 0.33 (SD = 0.08), 0.52 (SD = 0.09), 0.72 (SD = 0.12), 0.94 (SD = 0.08) for the 0%, 30%, 50%, 70%, and 100% categories, respectively. Figure 2A displays the distributions and confidence intervals of the different CNV thresholds when considering all chromosomes at different percentages of mosaicism (0%, 30%, 50%, 70% and 100%). The distribution obtained using all chromosomes is very similar to the distribution obtained when each chromosome was analyzed separately using the same method (Figure 2B). 

To check if our algorithm was correctly diagnosing percent mosaicism, 18 samples with different percentages of mosaicism (40%, 60% and 100%) generated using two cell lines with trisomy in chromosome 8 and 9 (GM00425 and NA09287, respectively) were analyzed. 100% of the samples amplified correctly (18/18), and 94.4% (17/18) were correctly categorized; only one 40% mosaic sample was mis-categorized as being high mosaic instead of low. All QC criteria were met (Table 1).

Finally, to estimate the percentage of mosaicism in TE biopsies, we applied the algorithm retrospectively to 14, 108 TE biopsies analyzed in 10 different diagnostic laboratories from our group. The overall percentage of mosaicism was 5%, with 3.66% (SD = 0.86) of samples classified as low-degree mosaicism and 1.34% (SD = 0.36) samples as high-degree mosaicism. The differences among laboratories were not significant (*p* < 0.05). These data are in concordance with previously reported percentages [21].

### 3.2. Phase II: PGT-SR 

For these samples, the amplification rate was 100% (48/48). Samples were sequenced in four 520 chips, with only 12 samples per run. The average number of reads per sample was 305,287 (156,970–604,356; SD = 120,052) and the MAPD mean value was 0.145 (0.106–0.3; SD = 0.037).

All deletions were detected (48/48), including the smallest, setting the detection limit to 5.6 Mb and making the concordance rate and sensitivity/specificity 100% (48/48) (Table 1).

## 4. Discussion

In recent years, several sequencing platforms have been applied to PGT-A, and initial publications [10,11,12,13,14,15] have highlighted the need for a proper validation of each platform, mostly for mosaicism levels and resolution for de novo and inherited Del/Dup. Here, we describe an improved, mostly automatized, fast, and accurate protocol for detecting whole uniform aneuploidies, de novo Del/Dup (≥10 Mb), unbalanced Del/Dup up to 6 Mb in carriers of structural rearrangements, as well as mosaic aneuploidies. A key advantage of NGS is that portions of the protocols can be automated minimizing the contamination risk, mismatch of samples, time, and cost. In this study, introducing automated library preparation with the Ion Chef™ increased the robustness and reproducibility of the NGS protocol. To our knowledge, this is the first study to extensively validate a semiautomated NGS protocol with the Ion Chef + S5 sequencer for PGT-A, PGT-SR (≥ 6 Mb), and mosaicism detection using a proprietary algorithm.

To validate our PGT-A strategy for detecting whole uniform chromosome aneuploidies and large Del/Dup, we used cell lines of known karyotypes. These cell lines have been utilized previously by other groups to validate PGT-A with both aCGH and NGS techniques [1], but, to our knowledge, our study was the most comprehensive regarding the number of different cell lines and different chromosomes affected (14 chromosomes divided between nine cell lines for whole uniform chromosome aneuploidy and six cell lines for large segmentals ≥ 10 Mb). Other authors, e.g., Kung et al. (2015) [1] and Goodrich et al. (2017) [28], used six and four cell lines, respectively, and only for whole-chromosome aneuploidies and without the benefit of the automation steps used in this study. 

Our PGT-A protocol was highly effective at detecting segmentals (≥ 10 Mb) in pools of 5-6 cells, mimicking TE biopsy. In 100% of samples, we not only correctly determined whole uniform chromosome aneuploidies, but also detected Del/Dup. These results are consistent with or improve upon those obtained in previous works, e.g., the Fiorentino et al. (2014) study [29], where authors set the size of de novo detectable segmentals to 14 Mb using the Illumina NGS platform. 

Our protocol was also highly effective at predicting mosaicism. We established thresholds for this using multiple mixtures of gDNA from cell lines with known karyotype, mimicking different mosaic percentages. Other groups have used distinct strategies combining different numbers of euploid and aneuploid individual cells [28,30]. Our gDNA approach allowed us to test mosaicism for more chromosomes than commercial cell lines (eight in total) can test, covering potential variability among chromosomes, since it is known that amplification can be biased by GC content [31]. Using the sequencing information from our samples, we developed a proprietary algorithm allowing the automated assignation of mosaic embryos to different categories, avoiding the subjectivity of both the scientist performing the diagnosis and the laboratory where the analysis has taken place. For this, we divided mosaic embryos into two categories: low and high. Such categorization is clinically relevant since implantation and ongoing pregnancy rates after the transfer of mosaic embryos relates to the degree of mosaicism, with low mosaic embryos exhibiting better implantation rates [17,20,24,32,33]. Nevertheless, euploid embryos should be chosen first for transfer, and the transfer of mosaic embryos should be coupled with pre- and post-genetic counselling, including the option of a new IVF cycle if there are no euploid embryos in the current cycle, to yield a better prognosis [34]. Segmental aneuploidies which are not uniformly present in blastocysts and have low predictive value in IVF/PGT-A [35,36] were not considered when determining mosaicism in embryos. 

Our algorithm to detect mosaicism is robust even when considering samples from different laboratories. We retrospectively analyzed the incidence of mosaicism in TE biopsies from 10 diagnostic Igenomix laboratories, finding an average incidence of 5% (3.6% low and 1.4% high), with no significant differences among laboratories, demonstrating the consistency of the algorithm. A wide range of mosaicism has been reported by different authors, suggesting that mosaicism may be over-diagnosed and highlighting the need to set thresholds for the degree of mosaicism that can be detected in a TE biopsy based upon the background signal that can interfere with the interpretation of results [37]. Interestingly, the different percentages of mosaic embryos reported in the bibliography are linked with the cutoffs used [38]. Some laboratories broadly defined mosaicism as being between 20% and 80% admixed aneuploid and euploid DNA and others, including us, used thresholds of 30% and 70%. Using the 20–80% range detected mosaicism in up to 17% of embryos, whereas the 30–70% threshold range decreased the mosaicism rate to 5%. Miscarriage rates are similar in both scenarios, indicating the 20–80% range may overdiagnoses mosaicism [38].

Finally, our protocol was successfully modified to create the PGT-SR protocol for detecting smaller Del/Dup (≥6 Mb). For this, we used four different cells lines with deletions in different chromosomes and with different breakpoints to assay the robustness of the technique in dealing with different chromosomal conditions. Other groups have reported the detection of deletions as small as 5 Mb in embryos using a similar platform [39]. However, these samples were amplified twice, first with Sureplex (Illumina, San Diego, CA, USA) and then during WGA, and therefore more DNA (100 g) than seen in regular protocols was used to prepare the libraries [39]. In an additional study, the authors reported automatic calling of deletions as small as 10 Mb but detected fragments around 5 Mb when data were examined manually, during which subjectivity could alter the diagnosis [40]. Additionally, the abovementioned studies all used TE biopsies not cell lines, likely contributing to the variation in the fragment sizes detected.

## 5. Conclusions

Our study indicates that the NGS platform Ion Chef plus S5 sequencer from ThermoFisher is a reliable tool for testing the chromosomal complement of preimplantation embryos, detecting whole uniform aneuploidies, segmentals (≥10 Mb), small rearrangements (Del/Dup ≥ 6 Mb), and degree of mosaicism. Part of the protocol is automated, remarkably reducing user error and the subjectivity often seen in manual PGT-A evaluation. Our automated algorithm allows for accurate, unbiased, and reproducible diagnoses for PGT-A and PGT-SR application. The next steps would be trying to enhance the detection of small rearrangements by improving the resolution to 6 Mb and moreover, to improve the accuracy of the diagnostic algorithm of mosaicism including data from the chromosomal analysis of the products of conception and livebirths.

## Figures and Tables

**Table 1:** Number of samples; informativity, and media of the quality parameters for all sample categories used in the validations.

TEST	Type of Sample	Total Samples	Informativity	Concordance Rates	Reads *	MAPD *	Duplicates
**PGT-A**	Uniform whole aneuploidies	96	100% (96/96)	100% (96/96)	173,053	0.170	10.00%
	Segmentals (≥ 10 Mb)	48	98% (47/48)	100% (47/47)	126,780	0.194	9.60%
	Mosaicism (0%, 40%, 60%, 100%)	18	100% (18/18)	94.4% (17/18)	148,874	0.174	6.63%
**PGT-SR**	Small rearrangements (≥ 6 Mb)	48	100% (48/48)	100% (48/48)	305,287	0.145	7.00%

* Mean of all samples for that category; MAPD: Median Absolute Pair-wise Difference
